# A Rare Case of Uterine Leiomyosarcoma with Metastasis to the Thyroid Gland

**DOI:** 10.1155/2020/8889843

**Published:** 2020-06-29

**Authors:** Gabriel Irizarry-Villafañe, Nadyeschka Rivera-Santana, Michelle Mangual-García, Alex González-Bóssolo, Rafael Trinidad-Hernández, Miosotis García-Maldonado, Víctor Carlo-Chévere

**Affiliations:** ^1^Endocrinology Department, San Juan City Hospital, San Juan, Puerto Rico 00936, USA; ^2^Building 1431 Ave Ponce de Leon, Suite 303, San Juan, Puerto Rico 00907, USA; ^3^HRP Labs, 300 Manuel Domenech Street, San Juan, Puerto Rico 00918, USA; ^4^Puerto Rico Pathology, 198 Calle Trinidad, San Juan, Puerto Rico 00917, USA

## Abstract

Uterine leiomyosarcomas are aggressive tumors associated with a poor prognosis. These neoplasms have high metastatic potential, more frequently affecting the lungs, liver, and peritoneum. There are very few cases of metastasis to the thyroid described in the literature. We present the case of a 47-year-old female diagnosed with uterine leiomyosarcoma metastatic to the thyroid gland. In this case report, we want to emphasize the utility of ancillary studies to help differentiate a leiomyosarcoma from anaplastic thyroid carcinoma since cytologic evaluation alone can be challenging.

## 1. Introduction

The thyroid gland is one of the most vascular organs in the body, but metastases to this organ are rare [1, 2]. Epidemiologic studies state that of all the thyroid malignancies, approximately 2% to 3% are secondary to metastasis [3]. As reported by Pastorello and Saieg, adenocarcinomas predominantly from the kidney, breast, and lungs and squamous cells carcinomas mostly from the head and neck account for the most common metastatic cancers to the thyroid [2].

Leiomyosarcomas, as well as benign leiomyomas, can coexist in the same uterus [4]. Uterine leiomyosarcoma (LMS) which originates from the smooth muscle of the uterus is a rare aggressive tumor with the propensity for distant metastasis [5, 6]. Uterine LMS metastasic to the thyroid gland is a rare event, accounting for 1% of the reported metastatic cases [7]. As per our knowledge, there have been only six reported cases of this malignancy with metastasis to the thyroid gland [1, 8].

It is well documented that thyroid metastases may mimic primary thyroid malignancies. Primary and secondary thyroid leiomyosarcoma can resemble anaplastic thyroid carcinoma (ATC), as well as medullary thyroid cancer (MTC) and melanoma, since they all can present as spindle cell tumors on cytology [2, 8]. We present this case of a metastatic uterine leiomyosarcoma to the thyroid to review the differential diagnosis of spindle cell neoplasms. We want to discuss the best approach to accurately identify a metastatic leiomyosarcoma to the thyroid during the fine needle aspiration cytology (FNAC) evaluation utilizing the pertinent ancillary studies.

## 2. Case Presentation

**Figure 1:**
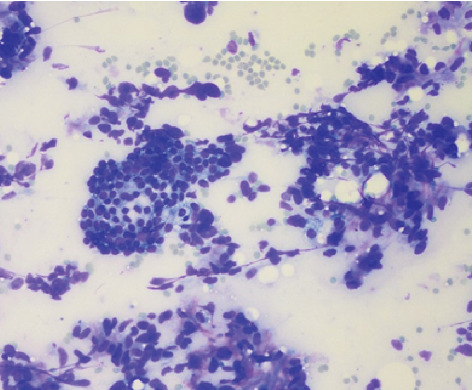
Thyroid nodule FNAB, Diff-Quick stain with marked cellularity of atypical spindle cells, dyscohesive, and in tissue aggregates with some binucleated cells.

**Figure 2:**
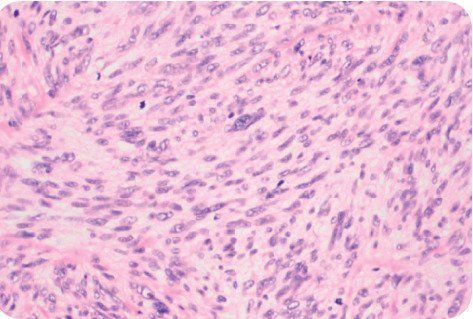
Postsurgical histology hematoxylin-eosin staining showed a spindle cell tumor with frequent mitotic figures.

**Figure 3:**
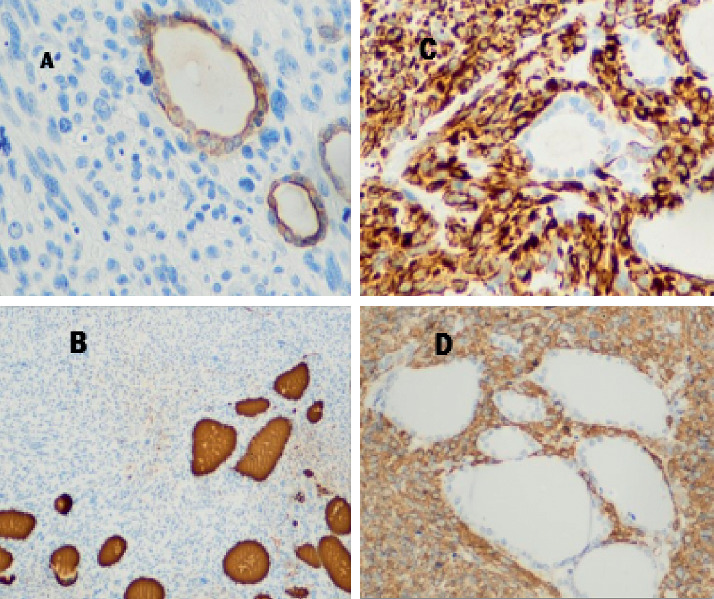
Immunohistochemical stains for (a) cytokeratin AE1/AE3 and (b) thyroglobulin showing entrapped thyroid follicles within fascicles of spindle tumor cells positive for (c) smooth muscle actin (SMA) and (d) desmin.

A 47-year-old female presented to the endocrinologist for evaluation due to a thyroid mass found on neck ultrasound ordered by her primary care physician due to neck discomfort. The patient had a medical history of myomatous uterus and microcytic hypochromic anemia secondary to abnormal uterine bleeding (AUB), with a negative endometrial biopsy. The patient denied radiation exposure to the head or neck nor a smoking history. The family history was noncontributory. Upon further interview, the patient denied obstructive symptoms such as shortness of breath, hoarseness, dysphagia, or odynophagia. Physical examination found neither goiter nor lymphadenopathy, but it was remarkable for a palpable left thyroid nodule. Thyroid ultrasound showed a left upper lobe 2.4 cm solid hypoechoic nodule with irregular borders. Due to sonographic findings of a high suspicious nodule, the patient underwent ultrasound-guided fine-needle aspiration biopsy. The Diff-Quick stain showed marked cellularity of atypical spindle cells, dyscohesive, and in tissue aggregates with some binucleated cells (Figure 1). Immunohistochemical (IHC) studies performed were reported as positive for thyroglobulin and thyroid transcription factor-1 (TTF-1) and negative for calcitonin. On the pathology report, these findings were consistent with poorly differentiated thyroid carcinoma. The diagnosis of anaplastic thyroid carcinoma was suggested based mainly on cytologic parameters. Because of these findings, a total thyroidectomy was performed promptly due to the poor prognosis associated with this diagnosis. After total thyroidectomy was performed, hematoxylin-eosin staining showed a spindle cell tumor with frequent mitotic figures (Figure 2). IHC studies evidenced the presence of normal thyroid follicles, which stained positive for thyroglobulin and cytokeratin AE1/AE3, entrapped within fascicles of atypical spindle tumor cells. The tumor cells stained positive for smooth muscle actin (SMA) and desmin (Figure 3) and negative for cytokeratin and thyroglobulin. A diagnosis of metastatic high-grade leiomyosarcoma was made. Also, the patient was found to have multiple left lung nodules found on imaging which were evaluated by CT-guided core needle biopsy with findings compatible with metastatic leiomyosarcoma. This was based on the Diff-Quick stain showing increased cellularity of atypical spindle cells which, on IHC studies, also stained positive for SMA and desmin and negative for TTF-1 and S-100.

Due to the patient's history of AUB, a total abdominal hysterectomy with bilateral salpingo-oophorectomy was performed. Pathology confirmed the uterine corpus as the primary site as results revealed a uterine leiomyosarcoma of 11 cm with microvascular invasion strongly positive for SMA, desmin, epithelial membrane antigen (EMA), and caldesmon. Due to the diagnosis of uterine leiomyosarcoma stage IVB, the patient received surgical management and chemotherapy treatment. Despite aggressive treatment, in addition to radiation therapy, the patient's disease has progressively spread to other organs including the liver and stomach.

## 3. Discussion

When evaluating a patient with a thyroid nodule, it is essential to consider metastasis among the differential diagnosis, whether symptomatic, asymptomatic, with a history of cancer, or the lack thereof. In our case, we portray a patient with no known history of malignancy that presented with metastasis to the thyroid gland. As stated by Nemenqani et al., clinical examination and radiographic imaging are not sufficient to distinguish between primary or secondary malignancies [8]. Secondary thyroid malignancies occasionally can imitate primary thyroid neoplasms, making fine needle aspiration cytologic diagnosis a challenging feat. The incorporation of ancillary studies to the FNAC is an important tool to achieve an accurate diagnosis.

Metastatic LMS and ATC are two entities that may have distinct clinical presentations although may be similar cytologically. ATC usually presents in older patients as an aggressive, rapidly enlarging thyroid mass with regional or distant spread. Uterine LMS, which is an aggressive neoplasm with a high tendency to metastasize, is most common in women over the age of forty and frequently presents with AUB [9]. As described in the literature, about 70% of patients with secondary thyroid malignancies presents with a palpable nodule [2]. Our patient was in her forties presenting with AUB of eight months of evolution and a palpable thyroid mass which should suggest a possible association between a uterine pathology and thyroid clinical findings. This underscores the importance of a detailed history and knowledge of the clinical presentation of different pathologies.

As described above, FNAC alone may be insufficient to distinguish primary vs. secondary malignancies of the thyroid. On cytology, ATC commonly can demonstrate “sarcoma-like” features presenting as a spindle cell tumor [10]. Ancillary studies have shown to be an effective tool in these puzzling situations. Thyroid differentiation immunomarkers such as transcription factor-1 (TTF-1) and thyroglobulin may be helpful [11]. However, an undifferentiated thyroid cancer such as ATC is less likely to stain positive for thyroglobulin or TTF-1, but most retain positivity for the transcription factor PAX-8 and the epithelial marker cytokeratin AE1/AE3 [12, 13]. The majority of leiomyosarcomas, as reported in the literature, stain positive for smooth muscle actin (SMA) and desmin [14]. It is pertinent to remember that medullary thyroid cancer can also present as a spindle cell tumor staining positive for TTF-1, keratins, and calcitonin [10].

In our case, the FNAC evaluation combined with a limited IHC study, which included thyroglobulin, TTF-1, and calcitonin, was not enough to differentiate between a LMS and a primary thyroid malignancy. As mentioned above, the FNAC was reported as positive in this high-grade tumor for these thyroid markers, except for calcitonin. These were falsely positive, due to staining of the normal thyroid follicular cells which were admixed with the neoplastic cells. Post-thyroidectomy histology marked positive for SMA and desmin but negative for TTF-1, CD34, cytokeratin AE1/AE3, and S-100 leading to the diagnosis of metastatic uterine LMS.

The FNAC is an excellent diagnostic tool for thyroid neoplasms. Nonetheless, in challenging cases such as this one, the IHC panel may be limited due to the scant cellularity that may be obtained in a cell block. Careful evaluation of the staining pattern and correlation with the morphologic findings of the staining cells is also of paramount importance to avoid false positives or false negatives, particularly in small biopsies such as FNA or core needle biopsies.

If the number of antibodies tested is felt to be insufficient for evaluation, a repeated FNA, or a core biopsy in selected cases, should be considered. A comprehensive approach, including both clinical and cytologic findings should be attempted, as each case will pose different challenges.

A detailed IHC panel should include SMA, desmin, cytokeratin pool AE1/AE3, and S-100 as a melanocyte marker combined with thyroid markers, in order to help distinguish a sarcoma from other spindle cell malignancies [2]. From the clinical point of view, some clues should be factored into the equation, for example, the presence or absence of the regional lymph node involvement. Since it is rare to find a high-grade primary thyroid malignancy without regional lymph node involvement, such a diagnosis without cervical adenopathy should alert to the possibility of a metastatic lesion. This knowledge may alert the pathology lab that additional markers are needed and may prompt the laboratory to request additional tissue.

Currently, there is no definite curative pharmacologic treatment for metastatic uterine leiomyosarcoma. Regarding the surgical treatment for distant metastatic leiomyosarcoma to the thyroid, there is a paucity of data. Nixon et al. describe that for patients with malignancies with localized small volume metastasis to the thyroid considered for surgical therapy, it may be managed with thyroid lobectomy with appropriate margins [15].

In conclusion, the diagnosis of metastasis to the thyroid gland can be difficult, and it is associated with poor prognosis [2]. Thyroid cancers most commonly are of a primary origin and of low-grade cytology [2, 6]. However, when confronting a high-grade thyroid lesion, this should prompt us to consider metastasis as the possible etiology of a thyroid nodule [2]. Spindle cell tumors on thyroid cytology can be due to anaplastic thyroid cancer, poorly differentiated thyroid cancers, MTC, and sarcomas, among other etiologies. We emphasize the importance of a detailed history and physical examination, knowledge of the differential diagnoses, and their presentations in addition to adequately accompanying FNAC with the pertinent ancillary studies in order to differentiate primary thyroid entities such ATC from metastatic spindle cell tumors such a leiomyosarcomas. There are scant data pertaining to the treatment of aggressive distant metastatic disease.
